# Treatment of Rickets

**Published:** 1893-03-04

**Authors:** 


### The Hospital Clinic.

[*The Editor will be glad to receive offers of co-operation and contributions from members of the profession. All letters should be addressed to* The Editor, The Lodge, Porchester Square, London, W.]

### PADDINGTON GREEN CHILDREN'S HOSPITAL.

### Treatment of Rickets (*continued*).

### III.—Medicines.

The chief medicine, and in many cases the only one employed, is cod liver oil, which ought rather to be classed amongst the food materials. It is usually dispensed in the form of an emulsion, which suits most palates. Half an ounce of the hospital emulsion contains two drachms of cod liver oil, and two grains each of hypophosphite of lime and hypophosphite of soda. The dose for an infant under nine months is half a teaspoonful, and after that age one or two teaspoonfuls. It is to be noted that the cod liver oil treatment is not only for the starved, rickety children, but is quite as much required by those who are over fat. If repeated doses of the drug produce nausea after food, one dose of a drachm of cod liver oil may be given at night and gradually increased. In ordinary cases the medicine is given three times a day immediately after food. The treatment must be prolonged, always for two or three months at least. In summer smaller doses are given than in winter, as the appetite often rebels against the oil in hot weather. In some cases the drug is better tolerated if the administration is interrupted that is to say, it is given for a week, and then stopped entirely for the following week, and so on. Some of the physicians prescribe phosphorus in doses of one hundredth of a grain, along with a drachm of cod liver oil. Other medicines are combined with the oil when there are special indications; thus, if anæmia is a marked symptom, syrup of the phosphate of iron is added; if there are digestive troubles, the fluid extract of malt; or, if there is a syphilitic taint, the syrup of the iodide of iron. For the specially troublesome period of teething mothers demand assistance. One grain of grey powder with five grains of bicarbonate of soda may be given at night, and three times during the day a teaspoonful of the following mixture: ℞ Calcis hypoph. gr. j. ½, tinct. nucis vomicae ♏j. ½, liq. calcis sacch. ♏viij, aquam ad. ʒj.

### IV.—The Treatment of Complications and Sequelæ.

1. *Diarrhœa.*—In summer the prominent rachitic manifestation for which children are brought to the hospital is diarrhœa, and this is usually due to the weakened condition of the intestinal mucous membrane, and the administration of unsound, or inappropriate food. The diet must therefore be carefully regulated in accordance with the principles given above, and the food must at first be very simple and digestible, and given more frequently and in smaller quantities than usual. The malt extract is combined with the milk, and half a teaspoonful of brandy four times a day may be added with advantage. A powder containing one or two grains of calomel is given to begin with, so as to clear out any fermenting materials in the intestine. Then cod liver oil is ordered, at first in small doses of ten or fifteen drops, and this is increased as rapidly as possible to the full amount. In cases of more prolonged and severe diarrhœa, it may be necessary to treat the catarrhal mucous membrane for a few days with a sedative mixture of bismuth and soda before commencing the cod liver oil. A warm flannel binder over the abdomen is very essential for rickety patients who are subject to diarrhœa.

2. *Bronchitis.*—This is the characteristic affection of the winter months. The milder cases are treated at home. The child must be put to bed, and kept there until the attack is over. Light curtains should surround the cot, which is to be placed near the fire, and a steam kettle is ordered to keep the atmosphere of the room moist, but not to surround the child with a dense mist. In out-patient practice it is found that poultices often do more harm than good, owing to the ignorance of mothers as to their preparation and application. A liniment is therefore ordered to be rubbed on to the back and front of the chest night and morning. The formula is as follows: Linim. terebinth. acet. three parts, linim. belladonnæ two parts, ol. olivæ one part. The chest must be thoroughly protected from cold. An oblong piece of wadding is taken of sufficient thickness, and a large hole is cut in the centre. It is then warmed, slipped over the child's head, and falls down on the back and chest. A flannel bandage or flannel undervest keeps the whole in position. The neck and the apices of the lungs are thus thoroughly protected, as well as the parts lower down. If there is much sweating, the wool must be changed as frequently as necessary, and a fresh warm piece applied. Cod liver oil is the best cough medicine in this variety of bronchitis. If there is pharnygeal cough, liquorice proves useful, and is much appreciated. The very acute cases are admitted into the hospital and treated on general principles.

3. *Convulsions.*—The more severe forms are usually associated with persistent intestinal disorder; the milder with some temporary local irritation, acting reflexly on the weakened nervous centres. All forms are serious, and must be energetically treated. For the more severe and persistent cases, chloroform is at once the speediest and the most certain means of relief. It is to be administered until the convulsive movements have ceased, and may be repeated if necessary. The inhalation of a few drops of nitrite of amyl acts promptly in some cases, but in others it has no effect, and is therefore not so reliable as chloroform. The inhalation of amyl is continued until the face is flushed, and must then be stopped. The fit having been checked, an enema of ten grains of bromide of soda is administered after the rectum has been emptied by a soap and water injection; or, if the child is able to swallow, five grains of the bromide are given by the mouth, and repeated in three hours. The effect is then maintained by continuing the medicine in four or five grain doses every four hours for a day or two. Bromide of potassium or soda is well borne in such cases by infants and children, and may therefore be pushed to a considerable extent. Chloral hydrate is sometimes combined with the bromide, in doses of from two to three grains; but its action must be carefully watched, as it is more dangerous. In the milder cases, where the convulsions are slight and occur at longer intervals, a hot bath, followed by skin rubbing so as to produce a rosy colour all over the body is employed, and the patient is kept quiet in bed. All possible sources of peripheral irritation are then looked to, such as diarrhœa or constipation, dysuria, inflammation of the middle ear, ascarides, &c., and are treated if present. Lancing of the gums for teething fits is seldom performed. A powder of one or two grains of calomel is commonly given, and this is followed by the administration of bromide of soda or potash in small doses to allay nervous irritability. In all cases the diet is carefully attended to. It must always be kept in mind that in rachitic children especially a convulsive seizure may mark the outset of a specific fever or other acute disease, and that consequently the child ought to be kept in bed as long as the temperature is at all above normal.

4. *Laryngeal Spasm.*—This is common in infants who are suffering from rickets. It is treated by the application of a hot fomentation, on which a teaspoonful of turpentine is sprinkled round the neck, and by the administration of an emetic of one grain of sulphate of copper in a teaspoonful of water. In older children, who are also the subjects of rickets, the spasm is usually accompanied by bronchial catarrh, and often occurs at the commencement of an attack of bronchitis, measles, or whooping cough. The patient is therefore kept in bed, and a soothing inhalation is ordered, such as a pint of boiling water containing one drachm of the compound tincture of benzoin.

5. *Deformities of Bones.*—The preventive treatment, while the bones are still soft, is here very important. The children must not be lifted by the arms, nor carried about with their limbs doubled up, nor carried at all by other children. If there is spinal weakness, the patient is kept lying on his back, and massage of the spinal muscles is employed twice a day, after he has been sponged all over with warm water. If he is beginning to walk, he must be kept entirely off his feet as long as the bones are soft. This is accomplished by the application of splints. Two wooden splints, well padded, are applied to each leg, one on the outside, and the other on the inside, and firmly bandaged. The splints must extend to the top of the thighs, and project for at least six inches below the feet. This will prevent his walking, but care must also be taken that he does not propel himself by crawling on his arms, or else they will get bent. The splint treatment is also employed for knock knees or bow legs as long as the bones are soft, but after they have become hardened, ostrotomy is the only treatment. For the softened and depressed ribs little can be done locally, and constitutional measures must be trusted to.

6. *Scurvy.*—This is a condition which, although not directly due to rickets, is not unfrequently present in rachitic patients, and requires treatment. In children who are suffering from rickets, traceable to the prolonged use of artificial and preserved foods, care is taken in directing their future diet that a sufficient quantity of an antiscorbutic, as well as of an antirachitic element is present. This is secured by the administration of fresh cow's milk, potatoes, and the juice of fresh fruits. In children in whom the signs of scurvy are present, more special treatment is required. If the gums are spongy and ulcerated, and the breath offensive, a mouth wash of sanitas fluid (one part in ten of water) is prescribed, and the gums are painted with a solution of glycerine of tannic acid (one part in three of water). If the limbs are swollen and tender, cold compresses are applied to them, and the child is kept absolutely at rest in bed so as to avoid the pain caused on movement. The most important part of the treatment, however, consists in the free administration of antiscorbutic foods. In the case of infants under nine months, fresh cow's milk, raw meat juice (two ounces daily in milk), and a teaspoonful of orange juice twice a day, are given; while older children have in addition mashed potato or potato soup (made with meat stock, carrots, and turnips), oranges, lemons, and other fresh fruits. The patients, as a rule, take to this diet ravenously, and therefore care must be taken not to push it too much at first, as it has been found in some cases to lead to sickness and diarrhœa. The improvement is usually very rapid and striking, and no other treatment is required.

